# Clinical and PSG Characteristics of Children with Mild OSA and Respiratory Events Terminated Predominantly with Arousal

**DOI:** 10.1155/2021/5549423

**Published:** 2021-06-07

**Authors:** Yunxiao Wu, Li Zheng, Panting Wu, Yufen Tang, Zhifei Xu, Xin Ni

**Affiliations:** ^1^Beijing Key Laboratory of Pediatric Diseases of Otolaryngology, Head and Neck Surgery, Beijing Pediatric Research Institute, Beijing Children's Hospital, Capital Medical University, National Center for Children's Health, Beijing 100045, China; ^2^Department of Otolaryngology, Head and Neck Surgery, Beijing Children's Hospital, Capital Medical University, National Center for Children's Health, Beijing 100045, China; ^3^Department of Respiratory Medicine, Beijing Children's Hospital, Capital Medical University, National Center for Children's Health, Beijing 100045, China

## Abstract

**Objective:**

To analyze the clinical and polysomnographic characteristics in children with mild OSA and respiratory events terminated predominantly with arousal.

**Methods:**

Children aged 3–10 yrs who had mild obstructive sleep apnea (OSA) were enrolled. All children underwent polysomnography, and patients' data were collected by using sleep-related breathing disorders (SRBD) questionnaire and OSA-18 quality of life questionnaire.

**Results:**

In total, five hundred and seventy-seven children were eligible. Children in arousal predominant group were younger and showed a lower rate of male and obesity. Compared with that of the nonarousal predominant group, the total arousal index, arousal index related to respiratory event, the percentage of NREM stage 1 (N1%), the fraction of respiratory events that were hypopnea, and the mean and minimum oxygen saturation in the arousal predominant group were significantly greater. The percentage of NREM stage 3 (N%), index of obstructive, central, mixed apnea, the fraction of respiratory events that were obstructive, and central and mixed apnea were significantly lower in arousal predominant group.

**Conclusion:**

Children with mild OSA in the arousal predominant group had specific characteristics, including younger age, lower rate of male and obesity, worse sleep architecture, higher rates of hypopnea events, and better oxygenation. This trial is registered with NCT02447614.

## 1. Introduction

Obstructive sleep apnea (OSA) is a condition characterized by episodes of complete or partial obstruction of the upper airway, associated with blood-gas changes and atypical sleep patterns which lead to neurocognitive and cardiovascular sequelae. The prevalence of OSA in children varies from 1% to 5% and the most common cause is adenotonsillar hypertrophy. Adenotonsillectomy (T&A) is the first line treatment especially for those with severe OSA [1, 2]. Management of children with mild OSA, however, remains controversial, since the pathophysiologic cause affecting the natural history of mild OSA is not well understood, giving rise to a need for further research on underlying mechanisms. Previous studies have shown that up to two-thirds of the children with mild OSA (AHI 1–5 episodes/hour) relieved as they get older [3]. Chervin et al. suggested that watchful waiting is a reasonable option in children with mild OSA who have low baseline apnea hypopnea index, normal waist circumference, and few symptoms (in particular, little snoring) [4]. Volsky et al. conducted a long-term follow-up for children with mild OSA and concluded that close observation can be made and no surgery needs to be performed temporarily for children with the score of OSA-18 being 60 or less [5]. In addition, children with OSA of similar severity and similar size of adenoid or tonsils could show different clinical faces [6]. All these facts indicate significant variability of the OSA phenotype. It suggested that we should not only focus on apnea and hypopnea index but also clinical manifestation, characteristics of sleep architecture, and respiratory regulation in order to understand pediatric OSA comprehensively, which will contribute to individualized treatment.

The majority of apneas and all hypopneas terminated with an arousal or a drop in oxygen saturation. Arousal is a protective factor that can protect children from severe hypoxia at night. However, recent studies from adults have shown that repeated arousals can lead to sleep fragmentation, which damages the sleep architecture, reduces the stability of breaths, and aggravates OSA [7]. In clinical practice, we found quite a number of children who had mild OSA with respiratory events terminated with arousal predominantly. Whether this type of children is a special type and has different prognosis remains to be determined. In addition, few studies have been conducted focusing on the clinical and PSG characteristics of children with OSA whose respiratory events are associated predominantly with arousal.

The aim of this study is to explore the characteristics of clinical manifestations, sleep architecture and respiratory events of children with mild OSA in two groups, and respiratory events terminated predominantly with arousal (arousal predominant group) versus respiratory events terminated with arousal and oxygen desaturation synchronously (nonarousal predominant group).

## 2. Material and Methods

### 2.1. Subjects

This was a prospective cohort study performed at the Sleep Center, Beijing Children's Hospital, from January to December in 2019. Children aged 3–10 years old with mild or moderate OSA due to hypertrophy of adenoid and/or tonsil were recruited. Children who had neuromuscular diseases, craniofacial anomalies, genetic diseases, and recent respiratory tract infections within 2 weeks prior to PSG were excluded. Informed consent was obtained from either of the children's parents. Assent was also obtained from children >8 years old. This study was approved by the Institutional Ethics Committee of Beijing Children's Hospital.

### 2.2. Anthropometry and Collection of Clinical Data

A physical examination including measurements of height, weight, and visual evaluation of the tonsils was completed during the evening prior to PSG testing. Body mass index (BMI) was adjusted for age and gender using the growth curve for Chinese children. A BMI >85^th^ percentile or >95^th^ percentile was considered as overweight or obese, respectively [8].

A fibrolaryngoscopy examination of the adenoids was performed by an ENT specialist in the outpatient clinic prior to the sleep study. The tonsil grading scale was proposed by Brodsky in 1989, and the guideline (reference citation [9]) [9] in 2019 still used this standard. The degree of adenoid obstruction was documented as a percent obstruction of the measured distance between the anterior and posterior surfaces of the nasopharynx with adenoid hypertrophy being defined as the percentage larger than 50% [10].

Family history of snoring was considered positive if one of or both the child's parents snore. We defined a child as having allergic rhinitis or otitis media if he/she had been diagnosed with these diseases in the past year by doctor in medical records. A child was considered declining in academic performance if he/she had got a worse grade in school than she did the previous semester.

### 2.3. Polysomnography

A standard overnight PSG was performed using Compumedics E-series (Compumedics, Australia), Alice 5 (Respironics, USA), or SOMNO screen Plus PSG + V5 system (SOMNO medics GmbH, Germany). During monitoring, the children were accompanied by one of their parents in the same room. Monitoring lasted >7 h for each child. The following parameters were measured: six-channel electroencephalograph (EEG) with bilateral frontal, central and occipital leads, electrooculography (EOG), electromyography (EMG) with submental electrodes, electrocardiogram, airflow measured through the nose via both nasal pressure cannula and thermistor, and respiratory effort measured by thoracic and abdominal inductive plethysmography. Oxygen saturation was measured by pulse oximetry via a finger probe. Two technicians and one pediatrician who were trained in sleep medicine, who were unaware of the children's clinical feature, interpreted the PSG. Sleep stages and respiratory events were scored on the basis of the criteria by the American Academy of Sleep Medicine manual in 2012 [11, 12]. Score a respiratory event as a hypopnea if the airflow drop by ≥30%, which lasts for ≥2 breaths and with *a* ≥ 3% oxygen desaturation or an arousal. Obstructive apnea/hypopnea index (OAHI) was defined as the average number of obstructive, mixed apnea, and hypopnea per hour of the total sleep time (TST). Oxygen desaturation index (ODI) was defined as the average number of oxygen saturation drop of ≥3% per hour. Arousal during sleep stage N1, N2, N3, or *R* was scored if there is an abrupt shift of EEG frequency including alpha, theta, and/or frequencies greater than 16 Hz (but not spindles) that lasted for at least 3 seconds, with at least 10 seconds of stable sleep preceding the change. Arousal was scored during REM only when a concurrent increase in submental EMG lasted for at least 1 second. Children with 1 ＜ OAHI ≤ 5 were defined as mild OSA [13]. Children with mild OSA were divided into two groups based on the ODI and ODI/OAHI ratio following our center's clinical practice. Arousal predominant group includes children with respiratory events terminated almost exclusively with arousal (ODI < 1.0 and ODI/OAHI < 0.5) while the others were assigned to the nonarousal predominant group (ODI < 1.0 and ODI/OAHI ≥ 0.5 or ODI ≥ 1.0).

### 2.4. Sleep-Related Breathing Disorders and OSA-18 Questionnaire

Face-to-face interviews were conducted by research assistants. Caregivers were asked to complete the Chinese-version SRBD questionnaire and OSA-18 questionnaire for the assessment of symptoms of sleep disordered breathing and its impact on patients' quality of life.

The SRBD questionnaire consisted of 22 items clustered in four factors, “Breathing,” “Sleepiness,” “Behavior,” and “Others,” which evaluated childhood sleep-related breathing disorder symptoms over the previous 6 months. One point was recorded if the answer was “yes”; otherwise, zero point was recorded. The scores on each dimension were summed up to produce a total sum score ranging from 0 to 22, with higher scores indicating more symptoms [14].

The OSA-18 questionnaire was validated in Mandarin. It consisted of 18 items which were divided into 5 domains including sleep disturbance, physical suffering, emotional distress, daytime function, and caregiver concerns. The domains of emotional distress and daytime function contained three items, and the other domains contained four. A point scale was used ranging from 1 to 7 to grade the relative severity of the problem addressed in each item. The scores on each item were summed up to produce a total sum score ranging from 18 to 126. The total sum score of quality of life was used for grading the impact of OSA on quality of life: small (score <60), moderate (score 60–80), and large impact (score >80) [15].

### 2.5. Statistical Analysis

All statistical analyses were performed by using JMP software, version 11.0. Kolmogorov–Smirnov test was used to determine normality of data distribution. Continuous variables were presented as means ± SD or medians (25^th^ percentile, 75^th^ percentile) depending on whether their distribution was normal or skewed. For comparisons of continuous variables between the two groups, Student's *t*-test or Mann–Whitney *U* tests were used as appropriate. For categorical variables, they were expressed as frequencies and a Chi-squared test was used for comparisons between the two groups. The differences in sleep stage between groups were adjusted for age and gender using a linear regression model. Two-tailed test with a *P* value <0.05 was considered to be indicative of statistical significance.

## 3. Results

### 3.1. Subject Characteristics

Five hundred and seventy-seven children were eligible for the study. The mean age was 5.7 yrs and 373 (64.6%) were male. Arousal predominant and nonarousal predominant group accounted for 40.0% and 60.0% of the population, respectively. Children in arousal predominant group were younger [5.4 ± 1.7 versus 5.9 ± 1.7, *t* = −3.688, *P* < 0.001] and had a lower rate of male [136(58.9%) versus 237(68.5%), *χ*^2^ = 5.612, *P*=0.018] and obesity [7(3.0%) versus 56(16.2%), *χ*^2^ = 24.644, *P* < 0.001]. The rate of tonsillar hypertrophy, adenoid hypertrophy, allergic rhinitis, otitis media, decline in academic performance, and family history of snoring was similar between the two groups (All *P* > 0.05) (Table 1).

### 3.2. Characteristics of PSG Parameters

Compared with nonarousal predominant group, total arousal index [8.2(6.7, 9.9) versus 7.3(5.5, 9.0), *Z* = −4.684, *P* < 0.001] and the percentage of NREM stage 1 of total sleep time in the arousal predominant group were significantly greater and the percentage of NREM stage 3 of total sleep time was significantly lower (All *P* < 0.001). The N1% and N3% still showed significant difference after adjusted for age and gender by using a linear regression model (All *P* < 0.001).

The index of obstructive apnea, central apnea, mixed apnea, and the fraction of respiratory events that were obstructive, central, and mixed apnea were lower while the fraction of respiratory events that were hypopnea was greater in arousal predominant group compared to that in nonarousal predominant group (All *P* < 0.001). The ODI was lower and the arousal index related to respiratory event was greater in arousal predominant group than that of nonarousal predominant group (All *P* < 0.001). When compared with nonarousal predominant group, the mean and minimum oxygen saturation was higher in arousal predominant group (All *P* < 0.001) (Table 2).

A comparison of the differences in respiratory events between genders showed that the OAHI was not significantly different between the two groups [females versus males, OAHI: 2.0(1.4, 3.2) versus 2.2(1.5, 3.1), *Z* = −0.840, *P*=0.401]. The ODI was lower and ArI-resp, mean and minimum SpO_2_ were higher in females as compared to the males [females versus males, ODI: 0.9(0.3, 2.1) versus 1.2(0.4, 2.4), *Z* = −2.187, *P*=0.029; ArI-resp: 2.0(1.5, 3.0) versus 1.7(1.2, 2.7), *Z* = −2.425, *P*=0.015; mean SpO_2_: 98(98, 98) versus 98(97, 98), *Z* = −2.207, *P*=0.027; SpO_2_ nadir: 93(91, 94) versus 92(89, 94), *Z* = −2.981, *P*=0.003].

### 3.3. Symptoms of Sleep Disordered Breathing and the Quality of Life

Children in arousal predominant group showed lower score for the question “Is your child overweight?” in “others” dimension, (0(0, 0) versus 0(0, 1), *Z* = −3.396, *P*=0.001). The score of breathing dimension, sleepiness dimension, and behavior dimension of SRBD questionnaire did not differ from that in nonarousal predominant group. There was no significant difference in each domain and total scores of OSA-18 quality of life questionnaire between the two groups (Table 3).

## 4. Discussion

The existence of different clinical manifestations and prognosis outcomes of similar severity OSA in children suggest that potentially there may be different physiological subtypes predisposing to this disorder. But unlike adults, physiological typing is not well studied in pediatric OSA. Based on consequences of respiratory events, this study explored whether children with respiratory events terminated predominantly with arousal have special clinical manifestations, sleep architecture, and sleep respiratory patterns. We found that children in arousal predominance group were younger and showed a lower rate of male and obesity. Compared with those in nonarousal predominant group, children in arousal predominant group had much worse sleep architecture, higher rates of hypopnea events, and better oxygenation.

### 4.1. Considerations of Grouping Split

In clinical practice, we found quite a number of children who had mild OSA with respiratory events terminated with arousal predominantly. Respiratory events of the others were secondary to hypoxia or arousal and hypoxia synchronize. Thus, the arousal index secondary to respiratory events may be equivalent in the above two kinds of children raises question that children whose respiratory events are dominated by arousal cannot be effectively separated. We use ODI < 1 and ODI/OAHI < 0.5 so as to ensure the group of respiratory events terminated with arousal predominantly include events is associated with only arousal as much as possible rather than oxygen desaturation.

### 4.2. Clinical Characteristics

Children in arousal predominant group were younger and had a lower rate of male and obesity compared to those in nonarousal predominant group. Previous literature has reported that pediatric OSA did not have a significant prevalence of gender [1]. In our current work, there was a gender-related predisposition between the two groups. The higher percentage of girls, lower ODI, and greater ArI-resp in the arousal predominant group as compared to the nonarousal predominant group may indicate that genders respond differently to respiratory events. In addition, further comparison of the characteristics of respiratory events between genders showed that the girls had lower ODI and higher ArI-resp and mean and minimum SpO_2_ comparing to boys. It might indicate that girls are more likely to terminate respiratory events with arousal rather than with an obvious drop of oxygen than boys. This finding suggests that sex differences in OSA may be present early on and is consistent with adult literature [16]. However, the mechanism of gender differences in respiratory regulation among children is unclear. A previous study in adult showed that protection from disordered breathing may be provided by the stimulatory effect of progesterone on ventilation in part because it sensitizes chemoreceptors to hypoxia and hypercapnia [17]. Thus, the respiratory events can be prevented or prematurely terminated with arousal caused by a minor hypercapnia without a decidable oxygen desaturation. In children, Yang reported that follicle-stimulating hormone was significantly lower in male than in female children OSA [18]. We speculated that chemoreceptors sensitivity to hypoxia and hypercapnia was blunted by decreased follicle-stimulating hormone in OSA boys, which may lead to reduced probability of arousal to terminate respiratory events and consequently induced a growing tendency to the drop of oxygen desaturation.

In this study, there was also an age-related predisposition which may be due to a mechanism associated with growth. Obesity plays an important role in ventilation regulation. When obese children lie down, higher pressure in the chest cavity can lead to alveolar collapse at the end of the expiratory period, affecting the exchange of blood-gas [19]. Systemic inflammation caused by obesity are negatively associated with ventilation response [20, 21], making obese population susceptible to oxygen drop when the flow is limited.

The score of SRBD questionnaire, which could evaluate sleep-related breathing symptom, sleepiness, behavior, and others such as enuresis, fatigue, difficulty in waking up, and growth retardation, is similar between the two groups. In terms of the effect on quality of life, which was estimated by the score of OSA-18 quality of life questionnaire, both arousal and nonarousal predominant group showed the same mild effect on quality of life. There was no statistical significance of the proportion of academic performance lag between the two groups.

### 4.3. Characteristics of Sleep Architecture

The sleep architecture in arousal predominant group showed more obvious sleep fragmentation associated with higher arousal index, augment of nonrapid eye movement sleep stage 1, and shrink of nonrapid eye movement sleep stage 3 after adjusting for age.

In the present study, frequent arousals due to restricted air flow prevented the children in arousal predominant group from falling into slow wave sleep. Destruction of slow wave sleep damages cognitive functions such as attention, executive processes, learning, and memory [22–24]. The aspects above were tested by questions in SRBD questionnaire, such as whether your child felt tired, whether your child's school performance declined, or questions related to sleepiness or attention deficit hyperactivity in this study. Unfortunately, no statistically significant difference was shown between the two groups. The explanation may be that cognitive function could not be evaluated comprehensively and sensitively by the subjective questions we used.

### 4.4. Characteristics of Respiratory Events

The collapse of the upper airway during sleep raises PCO_2_ or declines PO_2_ which can activate the upper airway dilator muscles to restore pharyngeal patency during sleep, which can protect against OSA [7].

In our study, the rates of apnea were lower in arousal predominant group compared to that in nonarousal predominant group, suggesting that arousal is a protective mechanism against more severe OSA, which is consistent with the result from Suzuki et al. [25]. However, data form adults showed that premature arousal may lead to repetitive apnea due to inadequate accumulation of respiratory stimuli to enable upper-airway muscle recruitment [26–28]. In this subset of patients with low arousal threshold involving OSA, strategies to raise the arousal thresholds to an appropriate level could potentially resolve OSA without causing major changes in overnight oxygen saturation [29, 30]. Whether it could be a new treatment option for children with respiratory events terminated predominantly with arousal is a question that requires more research as we did not evaluate the low arousal threshold in this study.

In addition, the fraction of hypopneas was significantly greater in arousal predominant group in the current study. Literature reported that higher fraction of hypopneas which represented less severe airflow obstruction indicated a better anatomy [31]. That was how we think about it. We considered that this group of children had a relatively better anatomy than that in nonarousal predominant group. So, we think that this group of children should have a comprehensive evaluation of anatomical structure including but not limited to the adenoids and tonsils. Furthermore, more follow-up evidence was needed to see whether the children in arousal predominant group would heal on themselves more likely with the development of airway and respiratory regulation as they grow older.

There were limitations in this study. Firstly, we did not differentiate hypopneas into obstructive and central or monitor CO_2_ during PSG for subjects, which would hinder more interesting founding between the two subtypes. Secondly, as a lack of long-term follow-up in the current study, there was no prognostic characteristics of both kinds of children. In future, treatments that target different mechanisms leading to OSA in individual patients might allow us to lower the AHI even further in selected populations.

## 5. Conclusion

Children with mild OSA in the arousal predominant group had specific characteristics of demographic data, sleep architecture, and respiratory patterns, including younger age, lower rate of male and obesity, worse sleep architecture, higher rates of hypopnea events and better oxygenation. It suggests that sex differences in OSA may be present early on. Since the better oxygen saturation and greater fraction of hypopneas were closely related to low arousal threshold and relatively better anatomy, role of low arousal thresholds and evaluation of airway need to be carefully assessed and the prognosis of follow-up needs to be obtained in this population of children.

## Figures and Tables

**Table 1:** Demographic and clinical data of children with mild OSA in two groups.

	Arousal predominant	Nonarousal predominant	*Z*/*t*/*χ*^2^	*P* value
*n*, %	231 (40.0%)	346 (60.0%)		
Age, yrs	5.4 ± 1.7	5.9 ± 1.7	−3.688	<0.001
Male, *n* (%)	136 (58.9%)	237 (68.5%)	5.612	0.018
Obesity, *n* (%)	7 (3.0%)	56 (16.2%)	24.644	<0.001
BMI	14.6 (13.7, 15.6)	15.3 (14.2, 17.2)	−5.045	<0.001
^*∗*^Tonsillar hypertrophy, *n* (%)	172 (74.5%)	256 (74.0%)	0.517	0.772
^*∗*^Adenoid hypertrophy, *n* (%)	180 (77.9%)	266 (76.9%)	0.182	0.913
^*∗*^Allergic rhinitis, *n* (%)	93 (40.6%)	135 (39.1%)	0.126	0.723
^*∗*^Otitis media, *n* (%)	5 (2.2%)	9 (2.6%)	0.095	0.758
^*∗*^Decline in academic performance, *n* (%)	8 (3.5%)	16 (4.7%)	0.437	0.511
^*∗*^Family history of snoring, *n* (%)	126 (55.0%)	215 (62.5%)	3.191	0.074

^*∗*^There is a small amount of missing data.

**Table 2:** Characteristics of PSG parameters of children with mild OSA in two groups.

	Arousal predominant *n* = 231	Nonarousal predominant *n* = 346	*Z*/*t*	*P* value
TST, min	473.4 ± 50.7	470.1 ± 45.1	0.829	0.407
SE, %	86.9 ± 7.4	86.8 ± 8.0	0.251	0.802
^*∗*^N1, %	12.7 ± 4.2	10.6 ± 4.2	5.862	<0.001
N2, %	48.4 ± 6.4	47.7 ± 6.3	1.395	0.163
^*∗*^N3, %	20.5 ± 5.0	23.0 ± 5.9	−5.328	<0.001
R, %	18.3 ± 3.7	18.7 ± 3.7	−1.150	0.250
OAHI, h^−1^	2.0 (1.5, 3.1)	2.2 (1.4, 3.2)	−1.220	0.223
OAI, h^−1^	0 (0, 0.1)	0.1 (0, 0.2)	−4.779	<0.001
Mean, s	12.5 (10.6, 14.5)	13.0 (10.9, 16.0)	−0.942	0.346
Longest, s	12.8 (12.0, 14.5)	14.0 (11.5, 19.0)	−0.437	0.662
CAI, h^−1^	0.5 (0.2, 0.8)	1.2 (0.7, 1.9)	−11.405	<0.001
Mean, s	12.0 (11.0, 14.0)	12.0 (11.0, 13.0)	−2.425	0.015
Longest, s	15.5 (13.5, 17.0)	17.0 (15.0, 19.5)	−1.761	0.078
MAI, h^−1^	0 (0, 0.1)	0 (0, 0.1)	−3.626	<0.001
Mean, s	16.0 (14.0, 20.3)	15.8 (12.5, 19.0)	−1.756	0.079
Longest, s	18.0 (16.5, 22.0)	17.0 (13.6, 21.0)	−0.904	0.366
HI, h^−1^	1.9 (1.4, 2.9)	2.0 (1.3, 2.9)	−0.034	0.973
Mean, s	19.4 (17.0, 22.0)	19.0 (16.0, 22.8)	-1.593	0.111
Longest, s	39.0 (31.0, 50.0)	37.0 (29.0, 46.0)	−2.033	0.042
Fraction respiratory events				
OA %	0 (0, 2.2)	0 (0, 4.7)	−4.088	<0.001
CA%	17.8 (7.7, 28.8)	36.4 (22.4, 50.0)	−10.305	<0.001
MA%	0 (0, 2.9)	0 (0, 4.6)	−2.812	<0.001
H %	76.9 (64.3, 88.5)	56.8 (44.4, 70.7)	−10.793	<0.001

ODI, h^−1^	0.3 (0.1, 0.5)	2.1 (1.3, 3.0)	−20.119	<0.001
^∗^ArI total, h^−1^	8.2 (6.7, 9.9)	7.3 (5.5, 9.0)	−4.684	<0.001
ArI-resp, h^−1^	2.1 (1.5, 2.9)	1.6 (1.1, 2.6)	−5.116	<0.001
Mean SpO_2_, %	98 (98, 98)	98 (97, 98)	−5.248	<0.001
SpO_2_ nadir, %	94 (93, 96)	91 (88, 93)	−14.888	<0.001

TST, total sleep time; SE, sleep efficiency; N1%, N2%, N3%, R%, percentage of NREM 1, 2, 3, and *R* sleep of TST; OAHI, obstructive apnea hypopnea index; OAI, obstructive apnea index; CAI, central apnea index; MAI, mixed apnea index; HI, hypopnea index; OAI%, CAI%, MAI%, HI%, fraction of respiratory events that were obstructive, central, mixed apnea or hypopnea; ODI, oxygen desaturation index; ArI total, total arousal index; ArI-resp, respiratory arousal index. ^*∗*^Group was still an independent factor for the dependent variable after controlling for age and gender using a linear regression model.

**Table 3:** Scores of SRBD and OSA-18 in children with mild OSA in two groups.

	Arousal predominant	Nonarousal predominant	*Z*	*P* value
	*n* = 231	*n* = 346		
Scores of SRBD questionnaire				
Breathing	3 (1, 5)	3 (1, 5)	−0.548	0.584
Sleepiness	0 (0, 0)	0 (0, 0)	−0.754	0.451
Behavior	1 (0, 4)	1 (0, 3)	-0.561	0.575
^*∗*^Others	0 (0, 0)	0 (0, 1)	−3.396	0.001
Total	5 (2, 8)	5 (2, 8)	−0.073	0.942

Scores of OSA-18				
Sleep disturbances	13 (10, 16)	13 (10, 16)	−0.028	0.978
Physical suffering	15 (13, 18)	15 (13, 18)	−0.058	0.954
Emotional distress	8 (6, 11)	9 (6, 12)	−0.461	0.645
Daytime function	9 (5, 11)	8 (6, 11)	−0.598	0.550
Caregiver concerns	19 (14, 22)	18 (15, 23)	−0.230	0.818
Total	62 (53, 75)	64 (53, 73)	−0.204	0.839

^*∗*^Only the score of question in “others” dimension “Is your child overweight?” was statistically significant (0(0, 0) versus 0(0, 1), *Z* = −3.396, *P*=0.001).

## Data Availability

The data are available upon request through e-mail to the corresponding author.

## Abbreviations

ArI: Arousal index
ArI-resp: Respiratory arousal index
BMI: Body mass index
CAI: Central apnea index
EEG: Electroencephalography
EMG: Electromyography
EOG: Electrooculography
HI: Hypopnea index
MAI: Mixed apnea index
OAI: Obstructive apnea index
OAHI: Obstructive apnea-hypopnea index
ODI: Oxygen desaturation index
OSA: Obstructive sleep apnea
PSG: Polysomnography
SE: Sleep efficiency
SRBD: Sleep-Related Breathing Disorders
T&A: Adenotonsillectomy
TST: Total sleep time.

## Additional Points

*Highlights.* (1) In the current study, children with mild OSA in the arousal predominant group had specific characteristics of demographic data, sleep architecture, and respiratory patterns, including younger age, lower rate of male and obesity, worse sleep architecture, higher rates of hypopnea events, and better oxygenation. (2) The result suggested that sex differences in OSA might be present early on. (3) Since better oxygen saturation and greater fraction of hypopneas were closely related to low arousal threshold and relatively better anatomy, role of low arousal thresholds and evaluation of airway need to be carefully assessed and the prognosis of follow-up needs to be obtained in children with respiratory events terminated predominantly with arousal.
